# Trajectories of mental health across the primary to secondary school transition

**DOI:** 10.1002/jcv2.12244

**Published:** 2024-05-16

**Authors:** Caitlyn Donaldson, Jemma Hawkins, Frances Rice, Graham Moore

**Affiliations:** ^1^ Centre for Development, Evaluation, Complexity and Implementation in Public Health Improvement (DECIPHer) School of Social Sciences Cardiff University Cardiff UK; ^2^ Wolfson Centre for Young People's Mental Health Cardiff University Cardiff UK

**Keywords:** latent class growth analysis, mental health, school transition, young people

## Abstract

**Background:**

Adolescence is a period of profound developmental change during which the prevalence of mental health problems starts to increase. It also typically coincides with a school transition. Understanding mental health trajectories through school transition is important to inform interventions to support young people's mental health during this period.

**Methods:**

In a longitudinal study with three assessment points spaced six months apart spanning the transition from primary (T1 = end of primary school [Year 6]) to secondary school (T2 = beginning of the first year secondary school [Year 7]; T3 = end of first year of secondary school [Year 7]) we carried out a latent class growth analysis of symptoms of common mental health problems. Young people (mean age at baseline of 11.2 years, standard deviation 0.29; 46.8% female; 53.2% male) from South East England (*n* = 1861) were included. We modelled emotional problems, conduct problems, hyperactivity and peer problems in parallel over the transition period. Individual‐level variables: socioeconomic status (SES), special educational need(s) (SEN), gender, negative life events (NLEs) and being worried about transition were tested as predictors of trajectory class membership using multinomial logistic regression.

**Results:**

A model with four trajectory classes provided the best fit to the data: ‘persistently elevated’ mental health problems, ‘emotional and peer problems’, ‘hyperactivity and conduct problems’ and ‘persistently low’ mental health problems. Class membership was differentially predicted by the individual‐level variables.

**Conclusions:**

Young people from low SES backgrounds, those with SEN and those who have experienced two or more NLEs are more likely to exhibit trajectories with elevated mental health difficulties through the transition to secondary school. Young people who were worried about transition were more likely to belong to a trajectory class characterised by elevated emotional problems.

## Graphical abstract

Using latent class growth analysis, this analysis found four trajectory classes of young people's mental health across the primary to secondary transition. These classes were predicted by individual‐level characteristics.

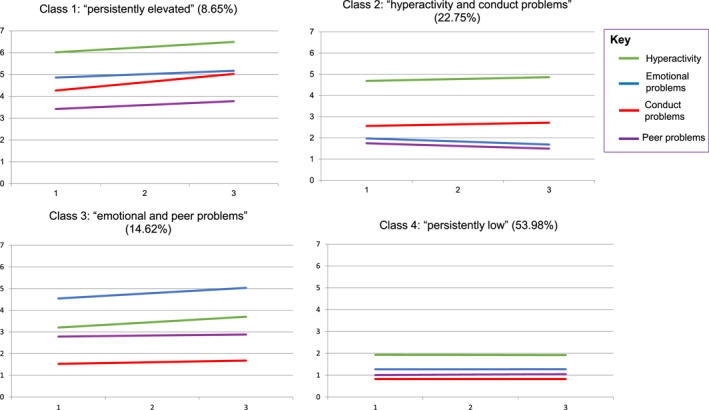

**Key points**

- This paper found four distinct mental health trajectory classes for young people experiencing the primary to secondary school transition at age 11.
- Three of these classes suggested elevated mental health symptoms in one or more domains of mental health.
- Membership of these trajectory classes was predicted by demographic characteristics including gender, socioeconomic status and special educational needs.
- This research builds on an increasing body of evidence of young people's mental health trajectories in early adolescence and those young people most at risk of poor mental health.

## INTRODUCTION

Mental health problems typically begin in adolescence or early adult life (Kessler et al., 2005) and are associated with impairment in educational and social functioning. While mental health symptoms may demonstrate stability within and between individuals, they are also frequently episodic and fluctuate over time (Oldehinkel & Ormel, 2023). Therefore, modelling the longitudinal development of mental health problems and their patterns of stability is informative. Some evidence suggests that early identification and provision of support for young people with emotional problems may be helpful for improving academic outcomes such as school attainment (e.g. Riglin et al., 2014).

Within trajectory research there are differences in approaches. While it is possible to split trajectories by individual characteristics, it is also possible to take a data‐driven approach. This considers whether there are distinct populations of individuals who share common trajectories within the same sample based solely on the data rather than a priori measured variables (Ram & Grimm, 2009). This approach may offer insights into subpopulations that would otherwise be overlooked and provide a more comprehensive understanding of trajectory groups.

Several studies have investigated mental health trajectories in young people using growth mixture modelling (GMM) approaches. Ellis et al. (2017), reviewed analyses of depressive symptoms trajectories and found that in all 18 studies identified, the largest subgroup consisted of those with low and stable depressive symptoms, while 14 (77%) had at least one group that had declining symptoms over time, and 14 (77%) had at least one group that had increasing symptoms over time. Nevertheless, comorbidity is common between mental health problems (Krueger et al., 2003) and certain groups of young people for example, those who are neurodiverse, are also more likely to exhibit mental health problems, yet the majority of studies of mental health trajectories do not consider this. Emotional, behavioural and peer problems and hyperactivity can coexist within time but also show different developmental patterns over time and outcomes may vary when different types of difficulty coexist (Richards & Abbott, 2009).

A number of studies have used a parallel process approach to mixture modelling. Shi and Ettekal (2021), for example, identified four parallel trajectory classes of internalising and externalising behaviours in children and young people assessed annually from age 6–17. These included a low risk group (22.8%), a chronic co‐occurring group (30.1%), moderate co‐occurring (28.5%) and pure externalising (18.6%). Similarly, McCoy et al. (2018) modelled four outcomes (internalising, externalising, attention and social difficulties) in parallel in children aged 4–10 years and also extracted four classes, which were described as ‘early recovery’ (7%), ‘late recovery’ (7%), ‘increasing attention and externalising problems’ (13%), and ‘low and stable’ (74%). Gender and ethnicity were differentially associated with class membership.

Not only is adolescence a period of profound developmental transition, it typically involves an important educational transition. Transition is a period of heightened susceptibility to adaptive or maladaptive changes (Rutter, 1989). Hence, transition periods may provide important opportunities at which interventions can be implemented, both because there may be greater sensitivity to new learning, and because engagement is likely to be higher due to the uncertainty and anxiety transition produces (Vitaro & Tremblay, 2008). In the UK, most young people transition from primary to secondary school at age 11. It can be a period of excitement alongside worry for young people (Moore et al., 2021; Rice et al., 2021) and up to a third of young people may find the process problematic (Waters et al., 2014).

Exploring trajectories of mental health across this period may help to better understand the subpopulations of young people most likely to experience poor mental health and to target interventions to support psychological wellbeing and transition to these individuals. Research shows that a number of factors may influence young people's transition to secondary school, including gender, socioeconomic status (SES), special educational needs (SEN) status, negative life events (NLEs) and being worried about transition. We briefly consider each of these in turn.

Gender has a strong influence on the mental health trajectories that young people follow, with significant differences in the shape, level and timing of onset for males and females (Gutman & Codiroli McMaster, 2020). Previous research has reported that females tend to worry more about the transition to secondary school (Rice et al., 2021).

Low SES is associated with higher rates of mental health problems (Yu & Williams, 1999). It is also associated with greater numbers of NLEs (Lantz et al., 2005) and within low income families, children who have experienced higher numbers of adverse childhoods experiences prior to school entry are more likely to experience adolescent trajectories of heightened socio‐emotional distress and low school bonding (Sanders et al., 2020). Children from lower SES backgrounds may perceive transition to be more stressful than children from more affluent families (Moore et al., 2021).

SEN is a broad term often encompassing learning and intellectual disabilities, specific learning difficulties such as dyslexia or dyspraxia, neurodevelopmental conditions and physical disabilities (Hughes et al., 2013). Children with SEN are frequently at higher risk of mental health problems than their peers (Bryant et al., 2020; Lai et al., 2019) and may have greater need for school support and targeted intervention over the transition period (Bunn & Boesley, 2019; Neal et al., 2016).

Stressors prior to transition are likely to impact on children's perceptions of transition to secondary school (Bonanno et al., 2007; Slavik & Croake, 2006). Children with SEN and with hyperactivity and conduct problems may also experience more NLEs (Powell et al., 2020). Indeed, hyperactivity can also make school a challenging environment for many young people, which may then result in poorer emotional outcomes (Capaldi, 1992).

Finally, higher concerns and social anxiety pre‐transition is associated with lower school belonging and higher loneliness 4 months after transition (Nowland & Qualter, 2020), and there is also a close relationship between perception of transition and future mental health (Zandstra et al., 2015). Bullying, friendships, academic pressures, a new physical environment and relationships with teachers are frequent causes of worry during transition (Moore et al., 2021).

Thus this analysis uses a parallel approach to model mental health trajectories over a key developmental transition. It aims to describe trajectories that consider multiple mental health problems at the same time, and to understand the association with key risk factors with a view to understanding which young people might merit special consideration for support around the transition to secondary school. The research questions posed are therefore:

1. Among young people experiencing a primary to secondary school transition, are there subpopulations that can be identified based on their trajectories of mental health difficulties?
2. Do the individual‐level variables considered (SES, SEN, gender, NLEs and being worried about transition) predict trajectory class membership?

## METHODS

### Sampling

The School Transition and Adjustment Research Study was conducted during 2012–2013. The sample consisted of young people transitioning from approximately 150 primary schools to nine secondary schools in South East England. Sampling was at the secondary school level and schools were selected because intake was representative of English and Welsh populations based on socioeconomic and SEN characteristics (Riglin et al., 2015). All young people due to attend the nine secondary schools were invited to participate. Data were collected from young people 6 months apart on three occasions—in year 6 (final year of primary school) and at the beginning and end of year 7 (first year of secondary school). At T1, 2161 pupils were invited to participate: 750 completed (34.7%); 108 withdrew; 1303 no response. At T2, 1960 students were invited: 1712 completed (87.3%); 137 withdrew; 111 absent. At T3, 1950 students were invited: 1653 completed (84.8%); 141 withdrew; 156 absent. Numbers of invitations fluctuated due to children moving into and out of sample schools. The analysis presented here includes students who had data for at least one mental health measure at one or more time points (*n* = 1861).

### Measures

The child‐reported version of the Strengths and Difficulties Questionnaire (SDQ) (Goodman, 1997) was administered at all three time points. Subscales (conduct problems, emotional problems, hyperactivity/inattention and peer problems) consist of five questions scored from 0 to 2. Prorated scores were used if more than half of each subscale was completed (Youth in Mind, 2016). Cronbach's alpha was calculated at each time point: emotional difficulties (.72–.74); conduct problems (.57–.62); hyperactivity (.69–.73); and peer problems (.56–.59). These are within the range reported as acceptable by Goodman (2001) and confirmatory factor analysis supported the four factor structure (Supporting Information S1: Appendix 1).

Correlations between each SDQ subscale were moderate, ranging from .24 to .59 (Supporting Information S1: Appendix 2). To categorise SDQ values, scores were rounded to the nearest integer and compared to the established and validated clinical cut‐point thresholds (low, slightly elevated, high and very high) of the SDQ (Youth in Mind, 2016). These vary by subscale, for emotional difficulties (0–4; 5; 6; 7–10); conduct problems (0–3, 4, 5, 6–10); hyperactivity (0–5, 6, 7, 8–10); peer problems (0–2, 3, 4, 5–10), respectively.

Secondary schools provided demographic data on child gender (female = 1), free school meal eligibility (FSM) (eligible = 1), ethnicity (White, Asian, Black, mixed ethnicity and other ethnicity), and first language. The term gender is pragmatically used as it is unclear whether schools reported based on biological sex or gender identity. FSM data was used as a proxy for SES, and is based on receipt of certain means tested benefits (UK Government, 2022). For SEN, responses were dichotomised (0 = no SEN and 1 = SEN at any level). The Life Events Checklist (Johnson & McCutcheon, 1980) was used to assess stress. Pupils were asked at T1 which of 19 NLEs they had experienced in the previous 12 months. These ranged from death of parent or sibling to doing badly at school. Students reported from 0 to 8 NLEs (Supporting Information S1: Appendix 3). Pupils were dichotomised into two groups based on the spread of responses: 0 = fewer than two NLEs; 1 = two or more NLEs. Children were also dichotomised based on their level of worry about transition to secondary school (1 = somewhat to very worried; 0 = not at all or little worried). Data were cleaned in STATA (version 15.1) and transferred to Mplus (version 8.6) for analysis.

### Analysis

#### Measurement invariance

Measurement invariance of the SDQ was tested to ensure consistent participant interpretation and responses over time (Putnick & Bornstein, 2016; van de Schoot et al., 2012). Three consecutive models were tested to assess: configural invariance (factor loadings, intercepts, item variances and correlations free to differ over time); metric invariance (equivalent loadings were fixed equal across time); strong invariance (equivalent loadings and intercepts were fixed equal across time). Good fit is indicated by root mean square error of approximation (RMSEA) values of <0.06; standardised root mean square residual (SRMR) values of <0.08; comparative fit index (CFI) values of >0.8 (Baumgartner & Homburg, 1996) and lower Bayesian information criterion (BIC) values. In equivalent groups with *n* > 300, non‐invariance is indicated by a change of ≤−0.010 in CFI, supplemented by a change of ≥0.015 in RMSEA or a change of ≥0.030 in SRMR (for metric invariance) or ≥0.010 in SRMR (for strong invariance) (Chen, 2007). Chi squared difference tests are also presented and evaluated.

#### Latent class growth analysis

Latent class growth analysis (LCGA) was used to identify classes of individuals with similar trajectories (Nagin, 1999). In an LCGA, within class variability is set to zero, making it a special case of a GMM (Wickrama et al., 2016). It was not possible to further extend the models to full GMMs as models would not converge. Emotional problems, conduct problems, hyperactivity and peer problems were modelled simultaneously and linear processes (Berlin et al., 2014). Due to high computational load involved in using a second order growth model, a composite measure was used for each subscale (Wickrama et al., 2016). Secondary school was included as a dummy covariate to adjust for clustering and modelled as time‐invariant (ICCs ranged from 0.00 to 0.02). Fit statistics for model selection were the bootstrap likelihood test (BLRT) (*p* < .05), Lo‐Mendell‐Rubin likelihood ratio test (LMR‐LRT) (*p* < .05), BIC and >5% of smallest class size (Wickrama et al., 2016). Pairwise Wald tests were carried out post‐hoc to test differences between classes.

### Three‐step procedure

The three‐step procedure (Asparouhov & Muthen, 2013) was carried out in line with Wickrama et al. (2016). Due to missingness in some predictors, MI was run in between the second and third steps (Muthen, 2012). The imputation model included gender, FSM eligibility, SEN status, being worried about transition, having two or more NLEs prior to transition, ethnicity (five categories) and first language (English/Non‐English). The secondary school ID variable and the dependent SDQ variables from each time point were included (He, 2010) and two hundred imputations run (von Hippel, 2020). The LCGA was re‐run with class means fixed using logit values obtained during the original LCGA to protect class membership and the Bolck‐Croon‐Hagenaars (BCH) approach used to apply weights reflecting measurement error of the latent class variable (Asparouhov & Muthen, 2021). Class was then regressed on the five predictors using multinomial regression to obtain log odds parameter coefficients (Wickrama et al., 2016) which were converted to odds.

### Missing data

Data at T1 were collected from children via post, resulting in higher levels of missingness across the four SDQ subscales (62%) than at T2–T3 where data were collected in school (18% and 14% respectively). Most students had demographic data on gender, FSM eligibility, SEN status, ethnicity and first language provided by schools. Both full information maximum likelihood (FIML) and multiple imputation (MI) were used to account for missing data. Students who did not participate at T1 were more likely to be male, from an ethnic minority background, eligible for FSM and to have SEN (Supporting Information S1: Appendix 4). As a sensitivity analysis, the LCGA was run using listwise deletion (*n* = 534) (Supporting Information S1: Appendix 5) (Jung & Wickrama, 2008). The four class solution had similar class proportions and entropy as in the FIML version, and the shape of the trajectories was also comparable (Supporting Information S1: Appendix 6). The three‐step procedure was also carried out using FIML but with complete case analysis for the regression (*n* = 660) (Supporting Information S1: Appendix 7). There were some differences in parameter estimates which likely arise from biases in the complete case data and further discussion is provided in the supplementary material. It provides some evidence to support using MI in this analysis.

## RESULTS

### Descriptive analysis

The mean age of respondents at T1 was 11.2 years (sd = 0.29); 53.2% were male and 46.8% female. Over half were White (59.8%), 23.2% were Asian, 7.5% were Black, 7.1% had mixed ethnicity and 2.4% were other ethnicity. Nearly three quarters of children spoke English as their first language (71.2%), 15.6% were eligible for FSMs and 18.1% had SEN. Approximately a third (31.1%) stated that they had experienced two or more NLEs prior to transition and 34.4% were worried about transition.

### Latent class growth analysis

Fit indices across the increasingly restricted models provided some evidence for measurement invariance (Supporting Information S1: Appendix 8). RMSEA changed by 0.001 from the configural to strong invariance model; SRMR by 0.003. CFI changed by −0.003 from configural to metric invariance model and then by −0.007 from metric to strong invariance. These changes are within the cut offs set by Chen (2007). The chi square difference tests however were significant (*p* < 0.001) at each step in the testing (change of 94.08 on 32 df from configural to metric invariance; 182.95 on 32 df from metric to strong invariance). This may indicate non‐invariance, but chi square statistics are also very sensitive to model changes when the sample size is >300, and this can result in reasonable models being rejected (Chen, 2007; van de Schoot et al., 2012). The other fit statistics (CFI, RMSEA, SRMR) are therefore typically more appropriate for assessing model fit, however, the criteria used are not perfect, and therefore the possibility of measurement non‐invariance remains (Putnick & Bornstein, 2016). The LCGA fit indices for the two, three, four and five class models are presented in Table 1. The four‐class model was selected as the best fit to the data. The BLRT remained significant for all models; the adjusted LMR‐LRT was only significant for the two class model, but was almost significant for the four class model.

**TABLE 1:** LCGA fit indices for 2–5 class models.

	2 Classes	3 Classes	4 Classes	5 Classes
LL (no of parameters)	−30,492.90 (29)	−30,122.03 (46)	−29,763.35 (63)	−29,608.20 (80)
BIC	61,204.14	60,590.39	60,001.02	59,818.70
Sample‐size adj BIC	61,112.01	60,444.25	59,800.87	59,564.55
Entropy	0.85	0.79	0.82	0.82
Adj. LMR‐LRT (*p*)	3059.79 (*p* < .0001)	735.99 (*p* = .24)	711.81 (*p* = .07)	307.90 (*p* = .69)
BLRT (*p*)	−32,034.75 (*p* < .0001)	−30,492.90 (*p* < .0001)	−30,122.03 (*p* < .0001)	−29,763.35 (*p* < .0001)
Class size (%)				
C1	1361.94 (73.18%)	248.33 (13.34%)	161.03 (8.65%)	950.59 (51.08%)
C2	499.06 (26.82%)	1100.19 (59.12%)	423.31 (22.75%)	100.35 (5.39%)
C3	‐	512.48 (27.54%)	272.04 (14.62%)	239.45 (12.87%)
C4	‐	‐	1004.62 (53.98%)	445.37 (23.93%)
C5	‐	‐	‐	125.24 (6.73%)

Abbreviations: BIC, Bayesian information criterion; BLRT, bootstrap likelihood ratio test; LL, log likelihood value; LMR‐LRT, Lo‐Mendell‐Rubin likelihood ratio test; No of parameters, number of estimated (freed) parameters.

**FIGURE 1:**
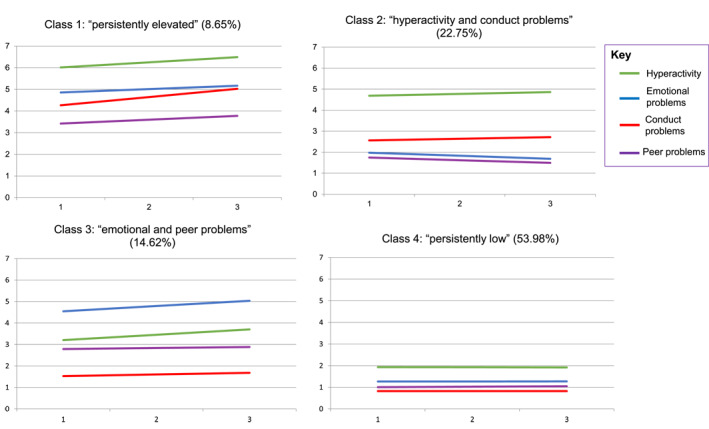
Class characteristics and percentage group membership. *X*‐axis represents each data collection point (time 1–3); *y*‐axis represents mean scores for each Strengths and Difficulties Questionnaire subscale within each class.

The four classes are represented in Figure 1. Table 2 presents mean values and categories. Table 3 provides pairwise Wald test statistics for subscales at T1.

**TABLE 2:** Mean values at each time point for each class.

	T1 mean	T2 mean	T3 mean	*p*‐Value for slope
Class 1 ‘persistently elevated’				
Emotional difficulties	4.86*	5.01*	5.17*	.56
Conduct problems	4.27*	4.65**	5.02**	.07
Hyperactivity	6.01*	6.25*	6.49*	.28
Peer problems	3.42*	3.60**	3.78**	.41
Class 2 ‘hyperactivity and conduct problems’				
Emotional problems	1.98	1.83	1.69	.12
Conduct problems	2.56	2.64	2.72	.42
Hyperactivity	4.69	4.77	4.86	.44
Peer problems	1.75	1.62	1.49	.09
Class 3 ‘emotional and peer problems’				
Emotional problems	4.55*	4.79*	5.04*	.20
Conduct problems	1.53	1.61	1.68	.39
Hyperactivity	3.21	3.45	3.70	.13
Peer problems	2.79*	2.84*	2.88*	.75
Class 4 ‘persistently low’				
Emotional difficulties	1.27	1.27	1.27	.96
Conduct problems	0.82	0.82	0.82	1.00
Hyperactivity	1.93	1.93	1.92	.86
Peer problems	1.01	1.03	1.05	.52

*Note*: Categories: * = Slightly raised; ** = High. All other values are within the ‘Close to average’ range.

**TABLE 3:** Post‐hoc pairwise Wald tests of each outcome variable at T1 between classes.

	C1 versus C2	C1 versus C3	C1 versus C4	C2 versus C3	C2 versus C4	C3 versus C4
Emotional difficulties	*F* = 30.42 *p* < .0001*	*F* = 0.22 *p* = .64	*F* = 48.17 *p* < .0001*	*F* = 51.86 *p* < .0001*	*F* = 14.89 *p* = .0001*	*F* = 114.70 *p* < .0001
Conduct problems	*F* = 12.75 *p* = .0004*	*F* = 50.13 *p* < .0001*	*F* = 67.82 *p* < .0001*	*F* = 12.26 *p* = .0005*	*F* = 59.84 *p* < .0001*	*F* = 13.98 *p* = .0002*
Hyperactivity	*F* = 8.99 *p* = .0027	*F* = 40.01 *p* < .0001*	*F* = 116.05 *p* < .0001*	*F* = 15.04 *p* = .0001*	*F* = 196.19 *p* < .0001*	*F* = 16.82 *p* < .0001
Peer problems	*F* = 16.19 *p* = .0001*	*F* = 1.51 *p* = .22	*F* = 36.12 *p* < .0001*	*F* = 10.40 *p* = .0013	*F* = 21.68 *p* < .0001*	*F* = 45.10 *p* < .0001*

*Note*: Bonferroni correction applied so that significance (*) is 0.05/24 = 0.0021. All tests have one degree of freedom.

Abbreviation: C, class.

Class 1 ‘persistently elevated’ (9% of students) was the smallest class and was characterised by individuals with relatively higher scores compared to those in the other classes for all four subscales. All four subscales were rated at ‘raised’ or ‘high’ levels across all time points and conduct problems and peer problems increased from ‘raised’ to ‘high’ across the transition period.

Twenty‐three percent of students belonged to class 2 ‘hyperactivity and conduct problems’. This class was represented by individuals with relatively low and decreasing levels of emotional and peer problems but relatively higher levels of hyperactivity and conduct problems. Conduct problems were significantly lower than in class 1, but significantly higher than in classes 3 or 4. Hyperactivity was significantly higher than classes 3 and 4, but not significantly lower than the high levels seen in class 1. All mean values were within the ‘close to average’ category, however, conduct problems and hyperactivity scores were at the top end of the range.

Class 3 ‘emotional and peer problems’ represented 15% of individuals and consisted of individuals with initially ‘slightly raised’ levels of emotional and peer problems. Conduct and hyperactivity scores were ‘close to average’. Mean emotional difficulties and peer problems were similar to those in class 1, conduct and hyperactivity levels were significantly lower than in class 1.

Finally, most students belonged to class 4 ‘persistently low’ (54%), where scores remained low and stable across all four subscales. Like class 2, all scores were within the ‘close to average’ SDQ category, but were significantly lower than the class 2 mean scores. Class 4 was used as the reference class in subsequent analyses.

### Associations with trajectory class

Odds ratios (ORs) and 95% confidence intervals (CIs) of associations between predictors and trajectory class are presented in Table 4. Female students had significantly lower odds of being in the ‘persistently elevated’ class (OR = 0.42; CI = 0.27, 0.66) and the ‘hyperactivity and conduct problems’ class (OR = 0.32; CI = 0.23, 0.45), but had significantly higher odds of being in the ‘emotional and peer problems’ class (OR = 1.84; CI = 1.25, 2.72). Students eligible for FSMs had higher odds of being in all three classes with elevated mental health difficulties compared to the ‘persistently low’ class, although the odds ratios were only significant for the ‘persistently elevated’ class (OR = 3.26; CI = 1.87, 5.67) and the ‘hyperactivity and conduct problems’ class (OR = 1.91; CI = 1.23, 2.98).

**TABLE 4:** Multinomial logistic regression models with class 4 as reference.

	Class 1 (8.65%) ‘persistently elevated’	Class 2 (22.75%) ‘hyperactivity and conduct problems’	Class 3 (14.62%) ‘emotional and peer problems’	Class 4 (53.98%) ‘persistently low’ (reference class)
	Odds ratios; [95% CI]; (% in class)			(% in class)
Female	0.42; [0.27, 0.66]; (33.97%)	0.32; [0.23, 0.45]; (30.24%)	1.84; [1.25, 2.72]; (63.60%)	(50.98%)
Eligible for free school meals	3.26; [1.87, 5.67]; (28.06%)	1.91; [1.23, 2.98]; (19.54%)	1.35; [0.75, 2.43]; (15.32%)	(12.13%)
Special educational needs	2.90; [1.71, 4.90]; (38.85%)	1.77; [1.15, 2.75]; (22.41%)	1.64; [0.97, 2.75]; (22.97%)	(11.92%)
Experienced 2 or more NLEs	4.45; [2.24, 8.84]; (55.00%)	2.07; [1.29, 3.33]; (40.14%)	3.01; [1.80, 5.03]; (51.49%)	(21.03%)
Worried about secondary school	3.05; [1.60, 5.82]; (52.50%)	1.17; [0.73, 1.87]; (31.47%)	3.35; [2.00, 5.59]; (54.46%)	(28.90%)

*Note*: % in class based on available data prior to multiple imputation; odds ratios calculated post‐multiple imputation.

Abbreviations: CI, confidence interval; NLEs, negative life events.

Students with SEN also had significantly higher odds of being in the ‘persistently elevated’ (OR = 2.90; CI = 1.71, 4.90) and ‘hyperactivity and conduct problems’ (OR = 1.77; CI = 1.15, 2.75) classes, compared to the reference class. The comparison between ‘emotional and peer problems’ and ‘persistently low’, while non‐significant, was in the expected direction. Students who had experienced two or more NLEs had higher odds of being in all classes with elevated mental health difficulties compared to the reference class, with a particularly strong effect in the comparison with the ‘persistently elevated’ class (OR = 4.45; CI = 2.24, 8.84). Finally, students who stated that they were worried about transition had significantly higher odds of being in the two classes characterised by high emotional problems (‘persistently elevated’ and ‘emotional and peer problems’).

## DISCUSSION

In a longitudinal study covering the transition period from primary to secondary school, we identified four distinct mental health trajectory classes. While most young people (54%) were situated within the ‘persistently low’ grouping, there remained a large proportion of young people who exhibited some elevated symptoms of mental health problems. For the ‘persistently elevated’ class, a particularly vulnerable group of young people experiencing elevated symptoms across the four subscales, class membership was predicted by all five covariates: FSM eligibility, SEN, being male, experiencing NLEs and being worried about transition. Some of this information on child characteristics will be known by primary schools and could be passed to secondary schools to support the transition process and inform transition interventions (Donaldson et al., 2022; Neal et al., 2016).

Notably, trajectories highlighted high levels of mental health difficulties for some young people by the end of primary school, underscoring the importance of intervening to support mental health prior to adolescence and to understanding the factors implicated in poor mental health in younger children. Recent analysis from Wales suggests that mental health difficulties increase through primary school and by the final year 29% of young people have elevated emotional difficulties and 15% elevated behavioural difficulties (Donaldson et al., 2023).

This analysis adds to previous research exploring how child mental health trajectories can be categorised, although sample differences make direct comparison difficult. As in other research, it suggested a four class solution, however there are differences in the shape of these trajectories. This is likely to be in part due to the relatively short time period and low number of data collections covered by this analysis. Shi and Ettekal (2021), for example, assessed from age 6–17 with 12 data collections, and while both this and their study had a ‘low’ grouping, a ‘high’ grouping, and a group with high externalising and low internalising difficulties, they were able to model non‐linear trajectories and improvements/decreases in mental health symptoms over adolescence. Similarly, McCoy et al. (2018) modelled four mental health outcomes in parallel with data collection started in pre‐school (mean age 4 years) and ending in fifth grade (at approximately age 10) with five points of data collection. The authors also extracted four classes but while their low and stable group is reflected in the findings here, it is more difficult to align the other groups which demonstrate recovery and increases in mental health difficulties over time. The samples also differed between studies—Shi and Ettekal (2021) selected children based on scoring below the median on a district literacy test; in McCoy et al. (2018) children were from low income backgrounds who were part of a school readiness intervention. The analysis presented here has a different focus to the other analyses by looking in detail at a short window of time as young people transition to a new secondary school, an important period of enhanced change and opportunity for intervention to support young people's mental health (Rutter, 1989; Vitaro & Tremblay, 2008). Future research to extend these findings within a UK context, starting at a younger age and continuing through adolescence would help further build context within a life course perspective around young people's mental health and school transition.

This study has a number of strengths. It is one of few studies to take a parallel process approach to modelling young people's mental health, and the only one to frame it from the perspective of school transition. However, it also has a number of limitations. Data were only collected at three points so only linear slopes could be estimated when alternative shapes may have been a more accurate reflection of young people's experiences. It is also not possible from this analysis to confirm the extent to which transition rather than typical developmental processes are impacting on trajectories—due to the linear nature of the analysis presented here, any discontinuities in mental health that occurred between T1 and T2 would be hidden by the linear slope. While there is evidence to suggest that transition does impact upon the mental health of some young people (Evans et al., 2018; Jindal‐Snape et al., 2020), this cannot be assumed from the findings presented here and more analysis is required. Future research should analyse trajectories across school systems with or without a transition to ascertain whether trajectories differ between the settings, and latent transition analysis could be used to estimate the impact of school transition on class membership (Nylund‐Gibson et al., 2022). This analysis relies on child‐report data and information from other informants (e.g. parents) would be particularly useful for hyperactivity and conduct problems (Loeber et al., 1991; Smith et al., 2000). Binarising the SEN variable may result in loss of detailed information on specific types of difficulty and it is possible that class membership may vary based on the type of difficulty.

Finally, there were missing data at the first data collection time point that required the use of FIML and MI for different stages of the analysis. The findings from MI differed from listwise deletion as reported in the supplementary material. Listwise deletion only provides unbiased estimates when data are missing completely at random (MCAR) (Newman, 2014), however, as observable characteristics predicted the pattern of missingness (Supporting Information S1: Appendix 4), this implies that the data were more likely to be missing at random than MCAR. Findings presented are therefore those obtained through MI, which is able to adjust estimates based on these observable characteristics and the estimates should therefore be less biased than in the complete case analysis. Missing data also meant it was not possible to account for primary school clustering, which may have artificially reduced confidence intervals on any statistical tests, although secondary school clustering was accounted for using dummy covariates and ICCs were low.

## CONCLUSION

This analysis suggests that there are four main subgroups of young people experiencing the transition to secondary school. Individual‐level characteristics predicted membership of trajectory classes, suggesting that young people from low SES backgrounds and those with SEN are more likely to have negative trajectories of mental health difficulties across the transition from primary to secondary school.

## AUTHOR CONTRIBUTIONS

**Caitlyn Donaldson**: Conceptualization; data curation; formal analysis; methodology; writing – original draft; writing – review & editing. **Jemma Hawkins**: Conceptualization; methodology; supervision; writing – review & editing. **Frances Rice**: Funding acquisition; methodology; supervision; writing – review & editing. **Graham Moore**: Conceptualization; methodology; supervision; writing – review & editing.

## CONFLICT OF INTEREST STATEMENT

The authors have declared that they have no competing or potential conflicts of interest.

## ETHICAL CONSIDERATIONS

The study was approved by the University College London research ethics committee. This secondary data analysis was approved by Cardiff University School of Social Sciences ethics committee (SREC/3652).

## Supporting information

Supporting Information S1

## Data Availability

The data that support the findings of this study are openly available in the UK Data Service at https://doi.org/10.5255/UKDA‐SN‐852714, reference number [852714].
