# Supplementary material for: Trajectories of mental health across the primary to secondary school transition
Source: JCPP Adv. 2024 May 16;5(1):e12244. doi: 10.1002/jcv2.12244 (PMC11889646; doi:10.1002/jcv2.12244)
Supplement: Supplementary file 1 — Supporting Information S1 [file JCV2-5-e12244-s001.docx]

Supplementary material

|  | Time 1 (n=711) | | | Time 2 (n=1548) | | | Time 3 (n=1613) | | |
| --- | --- | --- | --- | --- | --- | --- | --- | --- | --- |
|  | One factor | Two factors | Four factors | One factor | Two factors | Four factors | One factor | Two factors | Four factors |
| Chi-square test of model fit (df) | 1067.71 (170)  p<0.0001 | 646.50 (169)  p<0.0001 | 535.50 (164)  p<0.0001 | 1858.59 (170)  p<0.0001 | 1168.78 (169)  p<0.0001 | 919.42 (164)  p<0.0001 | 2533.79  (170) | 1402.07 (169) | 1079.34 (164)  p<0.0001 |
| Change in chi square (df) | - | 421.21 (1)  p<0.0001 | 111.00 (5)  p<0.0001 | - | 689.81 (1)  p<0.0001 | 249.36 (5)  p<0.0001 | - | 1131.72 (1)  p<0.0001 | 322.73 (5)  p<0.0001 |
| BIC | 21723.22 | 21308.57 | 21230.40 | 50516.46 | 49834.00 | 49621.36 | 52343.31 | 51218.97 | 50933.17 |
| Sample-size adjusted BIC | 21532.71 | 21114.88 | 21020.84 | 50325.86 | 49640.22 | 49411.69 | 52152.70 | 51025.19 | 50723.50 |
| RMSEA | 0.086 | 0.063 | 0.056 | 0.080 | 0.062 | 0.055 | 0.093 | 0.067 | 0.059 |
| CFI | 0.659 | 0.818 | 0.859 | 0.711 | 0.829 | 0.871 | 0.651 | 0.818 | 0.865 |
| SRMR | 0.075 | 0.056 | 0.051 | 0.069 | 0.054 | 0.049 | 0.079 | 0.056 | 0.050 |

Appendix 1: Fit indices for confirmatory factor analysis (CFA) of the four Strengths and Difficulties Questionnaire (SDQ) subscales at each data collection time point (T) for one, two and four factor solutions. The two factor solution was based on an internalising factor (consisting of emotional and peer problems subscales) and externalising problems (consisting of conduct problems and hyperactivity subscales). Bayesian information criterion (BIC); Root mean square error of approximation (RMSEA); comparative fit index (CFI); standardised root mean square residual (SRMR); degrees of freedom (df). Factors allowed to correlate.

|  | Emotional difficulties  (r) | Conduct problems  (r) | Hyperactivity  (r) | Peer problems  (r) |
| --- | --- | --- | --- | --- |
| Emotional problems | 1.00 |  |  |  |
| Conduct problems | 0.30, 0.35, 0.32 | 1.00 |  |  |
| Hyperactivity | 0.33, 0.38, 0.37 | 0.52, 0.55, 0.59 | 1.00 |  |
| Peer problems | 0.46, 0.44, 0.42 | 0.34, 0.36, 0.30 | 0.28, 0.25, 0.24 | 1.00 |

**Appendix 2: Pearson’s r correlations between each SDQ subscale at each time point – T1/T1, T2/T2, T3/T3 following FIML. Values varied only marginally when calculated without FIML using listwise deletion.**

| Number of NLEs | Freq (%) |
| --- | --- |
| 0 | 267 (37.55%) |
| 1 | 223 (31.36%) |
| 2 | 127 (17.86%) |
| 3 | 47 (6.61%) |
| 4 | 26 (3.66%) |
| 5 | 12 (1.69%) |
| 6 | 4 (0.56%) |
| 7 | 4 (0.56%) |
| 8 | 1 (0.14%) |

Appendix 3: Frequency of negative life events (NLEs) reported out of 19 possible NLEs.

|  | Characteristics of each subsample | | | | |
| --- | --- | --- | --- | --- | --- |
| Sample | Female  (n, mean, sd) | Eligible for FSM  (n, mean, sd) | SEN status  (n, mean, sd) | Ethnic minority  (n, mean, sd) | Non-English first language  (n, mean, sd) |
| All available data | (n=1860)  0.47 (0.5) | (n=1599)  0.16 (0.36) | (n=1598)  0.18 (0.39) | (n=1563)  0.40 (0.49) | (n=1599)  0.29 (0.45) |
| Missing SDQ data at T1 | (n=1150)  0.45 (0.5) | (n=938)  0.17 (0.38) | (n=937)  0.20 (0.40) | (n=912)  0.42 (0.49) | (n=938)  0.29 (0.45) |
| Not missing SDQ data at T1 | (n=710)  0.49 (0.5) | (n=661)  0.13 (0.34) | (n=661)  0.16 (0.36) | (n=651)  0.38 (0.49) | (n=661)  0.28 (0.45) |

Appendix 4: Missing data patterns for Time 1 in year 6 (final year primary school). FSM = free school meals; SEN = special educational needs; SDQ = strengths and difficulties questionnaire; sd = standard deviation. All variables are treated as binary. Ethnicity is used as a binary variable of White and ethnic minority.

|  | 2 Classes | 3 Classes | 4 Classes | 5 Classes |
| --- | --- | --- | --- | --- |
| LL (No of parameters) | -12372.30 (29) | -12184.99 (46) | -12008.15 (63) | -11935.46 (80) |
| BIC | 24926.73 | 24658.88 | 24411.97 | 24373.35 |
| Sample-size adj BIC | 24834.67 | 24512.86 | 24211.99 | 24119.41 |
| Entropy | 0.90 | 0.85 | 0.88 | 0.90 |
| Adj. LMR-LRT (p) | 1225.62 (p=0.03) | 371.14 (p=0.42) | 350.39 (p=0.18) | 144.03 (p=0.24) |
| BLRT (p) | -12990.85 (p<0.0001) | -12372.30 (p<0.0001) | -12184.99 (p<0.0001) | -12008.15 (p<0.0001) |
| Class size (%) C1 | 419.60 (78.58%) | 67.61 (12.66%) | 37.06 (6.94%) | 290.78 (54.45%) |
| C2 | 114.40 (21.42%) | 321.04 (60.12%) | 146.96 (27.52%) | 31.17 (5.84%) |
| C3 | - | 145.35 (27.22%) | 57.09 (10.69%) | 34.21 (6.41%) |
| C4 | - | - | 292.88 (54.85%) | 144.35 (27.03%) |
| C5 | - | - | - | 33.49 (6.27%) |

Appendix 5: Sensitivity analysis. Fit statistics for LCGA using listwise deletion (N=534)


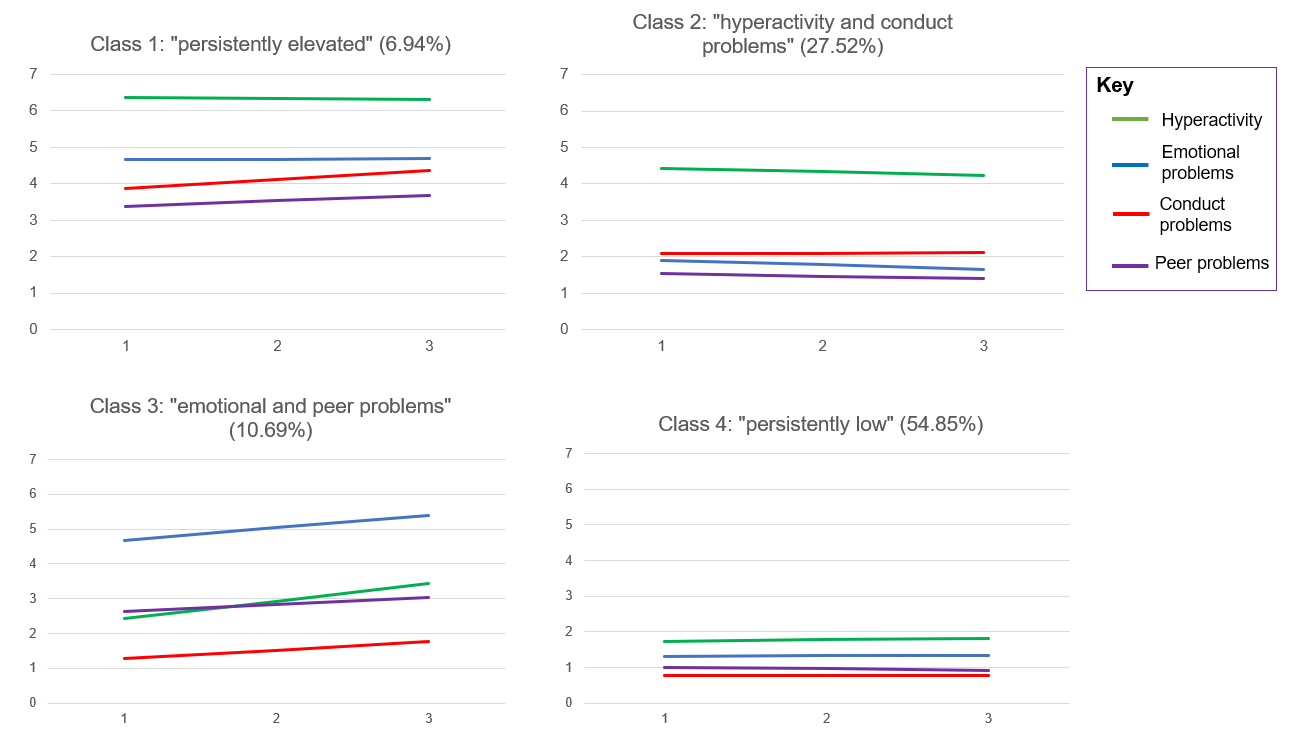


Appendix 6: Class characteristics and percentage group membership from sensitivity analysis (using listwise deletion for latent class growth analysis). X-axis represents each data collection point (time 1-3); y-axis represents mean scores for each SDQ subscale within each class.

|  | Class 1  “persistently elevated” | Class 2  “hyperactivity and conduct problems” | Class 3  “emotional and peer problems” | Class 4  “persistently low” |
| --- | --- | --- | --- | --- |
|  | Odds ratios [95% CIs] | | |  |
| Female | 0.77 [0.34, 1.73]  (48.57%) | 0.34 [0.19, 0.61]  (32.12%) | 1.40 [0.76, 2.58]  (60.64%) | (52.03%) |
| Eligible for free school meals | 2.60 [0.97, 6.94]  (22.86%) | 1.21 [0.54, 2.69]  (14.60%) | 1.33 [0.50, 3.50]  (14.89%) | (12.18%) |
| Special educational needs | 1.83 [0.62, 5.39]  (22.86%) | 3.03 [1.57, 5.83]  (24.82%) | 2.49 [1.20, 5.16]  (24.47%) | (9.90%) |
| Experienced 2 or more NLEs | 5.25 [2.26, 12.19]  (54.29%) | 3.09 [1.77, 5.40]  (40.15%) | 5.31 [2.89, 9.77]  (52.13%) | (20.05%) |
| Worried about secondary school | 3.45 [1.42, 8.36]  (54.29%) | 1.02 [0.56, 1.84]  (29.93%) | 3.25 [1.77, 5.96]  (54.26%) | (28.43%) |

Appendix 7: Multinomial logistic regression using complete cases within the FIML LCGA model. Odds ratios (ORs), 95% confidence intervals (CIs) (N=660). % in each class based on the 660 cases.

The parameters differ to some extent from those of the FIML analysis presented in the main body of the paper, and it is likely that using complete case analysis has introduced bias. For example, in the full 1861 sample just one student was missing data on gender (Appendix 4), and therefore the proportion of female students in each class was almost unaffected by missingness (class 1: 33.97% were female; class 2: 30.24%; class 3: 63.37%; class 4: 50.98%). This allowed the parameter estimate to be accurately calculated when multiple imputation was used to account for missingness in other covariates. However, using complete case analysis resulted in disproportionate loss of male students from class 1 (48.57% of the students remaining were female). This is reflected in the parameter estimates for female, with the findings non-significant in the complete case analysis and a shift in the parameter estimate (albeit the effect remains in the same direction). A similar effect is seen for the SEN variable in class 1, where the complete case analysis resulted in the disproportionate loss of students with SEN (from 38.85% when all students with data are included to 22.86% in the complete case analysis). The reason why more students with these characteristics were lost from the complete case analysis is largely because they were less likely to have data on negative life events and being worried about secondary school – i.e. they were less likely to complete the survey at T1. This suggests that missing data at T1 is, to some extent, missing at random (MAR) – i.e. it can be predicted by other characteristics within the data – the bias which MI can help to address.

|  | Chi-square, df | Change in Chi-square, df | CFI | RMSEA | Change in RMSEA | SRMR | Change in SRMR | BIC | Sample-size adjusted BIC |
| --- | --- | --- | --- | --- | --- | --- | --- | --- | --- |
| Configural invariance (null model) | 3822.31, 1584 | - | 0.887 | 0.027 | - | 0.047 | - | 120413.49 | 119441.33 |
| Metric invariance | 3916.39, 1616 | 94.08, 32 (p<0.001) | 0.884 | 0.027 | 0.000 | 0.049 | 0.002 | 120266.02 | 119395.52 |
| Strong invariance | 4099.34, 1648 | 182.95, 32  (p<0.001) | 0.877 | 0.028 | 0.001 | 0.050 | 0.001 | 120207.42 | 119438.58 |

Appendix 8: Measurement invariance indices. Fit indices: CFI = comparative fit index; RMSEA = root mean square error of approximation; SRMR = standardised root mean square residual; BIC = Bayesian information criterion.
